# IL-33 attenuates the development of experimental autoimmune uveitis

**DOI:** 10.1002/eji.201444671

**Published:** 2014-10-18

**Authors:** Mark Barbour, Debbie Allan, Heping Xu, Cheng Pei, Mei Chen, Wanda Niedbala, Sandra Y Fukada, Anne-Galle Besnard, Jose C Alves-Filho, Xiaoguang Tong, John V Forrester, Foo Yew Liew, Hui-Rong Jiang

**Affiliations:** 1 Strathclyde Institute of Pharmacy and Biomedical Sciences, University of StrathclydeGlasgow, UK; 2 Centre for Experimental Medicine, Queen's University BelfastBelfast, UK; 3 Institute of Infection, Immunity and Inflammation, University of GlasgowGlasgow, UK; 4 Institute of Medical Science, University of AberdeenUK; 5 Centre for Ophthalmology and Visual Science, The University of Western AustraliaAustralia; 6 Centre for Experimental Immunology, Lions Eye InstituteNedlands, Australia; 7 CEGMR, King Abdulaziz UniversityJeddah, Saudi Arabia; 8 Institute of Biology and Medical Sciences, Soochow UniversitySuzhou, China

**Keywords:** Experimental autoimmune uveitis, Inflammation, Interleukin-33, Macrophages, T cells

## Abstract

Interleukin-33 (IL-33) is associated with several important immune-mediated disorders. However, its role in uveitis, an important eye inflammatory disease, is unknown. Here, we investigated the function of IL-33 in the development of experimental autoimmune uveitis (EAU). IL-33 and IL-33 receptor (ST2) were expressed in murine retinal pigment epithelial (RPE) cells in culture, and IL-33 increased the expression of *Il33* and *Mcp1* mRNA in RPE cells. In situ, IL-33 was highly expressed in the inner nuclear cells of the retina of naïve mice, and its expression was elevated in EAU mice. ST2-deficient mice developed exacerbated EAU compared with WT mice, and administration of IL-33 to WT mice significantly reduced EAU severity. The attenuated EAU in IL-33-treated mice was accompanied by decreased frequency of IFN-γ^+^ and IL-17^+^ CD4^+^ T cells and reduced IFN-γ and IL-17 production but with increased frequency of IL-5^+^ and IL-4^+^ CD4 T cells and IL-5 production in the draining lymph node and spleen. Macrophages from the IL-33-treated mice show a significantly higher polarization toward an alternatively activated macrophage phenotype. Our results therefore demonstrate that the endogenous IL-33/ST2 pathway plays an important role in EAU, and suggest that IL-33 represents a potential option for treatment of uveitis.

## Introduction

Uveitis, particularly posterior uveitis, is a sight-threatening intraocular inflammatory disease, it can occur in isolation or associated with other systemic immunological diseases such as Vogt–Koyanagi–Harada disease and Behçet's disease. Experimental autoimmune uveitis (EAU) in the mouse closely resembles the pathology of human posterior intraocular inflammatory conditions such as sympathetic ophthalmia, sarcoidosis, and birdshot retinochoroidopathy 1,2. The pathology of EAU is mediated by autoantigen-specific CD4^+^ T cells whose activation is accompanied by the infiltration of tissue damaging leukocytes, such as macrophages and neutrophils, into the retina/choroid 3. Both Th1 and Th17 cells are thought to be responsible for the disease 4 while Th2 and regulatory T cells can protect against EAU 5. Previously, we have reported different phenotypes of macrophages in the retina tissues during disease progression from peak to later stages of EAU 6. While some macrophages, e.g. sialoadhesin positive macrophages, can exacerbate the inflammatory response in EAU 3, other types of macrophages are associated with suppression of the disease 3,7. Here, we have investigated the role of IL-33 8, the recently identified type 2 cytokine, in EAU and the effect of IL-33 on the phenotypic changes of T cells and macrophages during the disease development.

IL-33 is a member of the IL-1 cytokine family, which also includes IL-1α, IL-1β, IL-18, and IL-1Ra. Different from IL-1 and IL-18, full length IL-33 is bioactive and is released through cell necrosis 9 or via vesicles in endothelial cells and fibroblasts 10. IL-33 signals via the heterodimeric receptor consisting of ST2 and IL-1R accessory protein 11. The membrane form of ST2, encoded by the *ST2* gene 12, is expressed on cells including activated Th2 cells 13 and mast cells 14. *ST2* is alternatively spliced to produce a soluble form (sST2), which acts as a decoy receptor of IL-33 15. Human IL-33 was detected in epithelial cells, fibroblasts 8, and endothelial cells of inflamed tissues from patients with rheumatoid arthritis and Crohn's disease 16. In rodents, *Il33* mRNA was detected in various tissues and organs including lungs, lymph nodes, spleens, and the CNS 8. IL-33 has pleiotropic effects on immune responses 17 and plays an important role in the development of many immune-mediated diseases. Previous studies using in vivo disease models have shown that IL-33 exacerbates allergic inflammation 18 but confers resistance to parasite infection 19 and attenuates atherosclerosis 20 through inducing type 2 immune responses. However, IL-33 is also able to induce inflammatory hypernociception 21 and is a proinflammatory cytokine in both antigen- and autoantibody-induced arthritis via activating mast cells 17,22,23. Recent studies show that the IL-33/ST2 pathway plays a significant role in the amplification of alternatively activated macrophage (M2) polarization. M2 appears to play a key role in IL-33-mediated exacerbation of airway inflammation 24, protection against obesity-related metabolic events 25, and attenuation of EAE 26.

IL-33 has been detected in normal conjunctivae and human vascular endothelial cells of giant papillae formations in allergic conjunctiva 27. However, the role of IL-33 in the development of retinal inflammatory diseases is hitherto unknown. In the present study, we show that IL-33 protein is highly expressed in retinal cells, and the expression is markedly upregulated in EAU mice. Furthermore, we found that ST2-deficient (ST2**^−^**^/^**^−^**) mice developed more severe retinal inflammation compared with WT mice, while treatment of IRBP_1–20_-immunized mice with recombinant IL-33 significantly attenuated the severity of EAU. The reduced retinal inflammation of IL-33-treated mice was associated with decreased frequency of IFN-γ^+^ and IL-17^+^, but increased IL-5^+^ and IL-4^+^ CD4 T cells in the draining lymph node (DLN) and spleen. In addition, macrophages from the IL-33-treated EAU mice showed greater polarization toward the M2 phenotype. Together our results demonstrate that IL-33 is an endogenous regulator of retinal inflammatory disease and suggest that IL-33 may be a novel therapeutic agent for uveitis.

## Results

### Expression and function of IL-33 in the RPE cells

**Figure 1:**
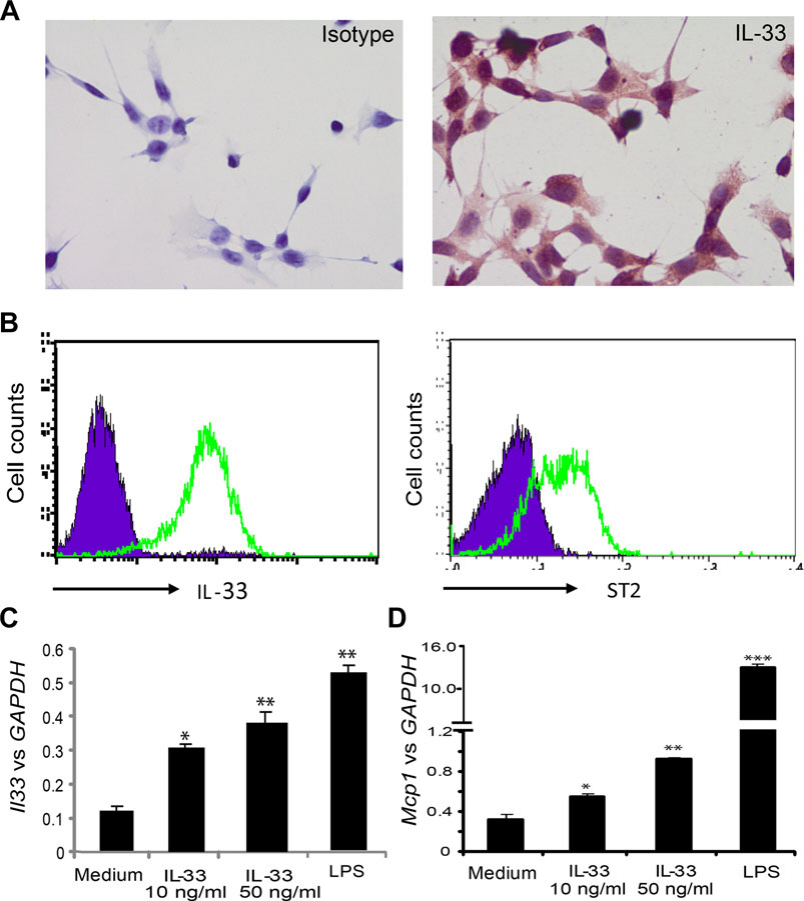
Expression and function of IL-33 on a murine RPE cell line. (A) Immunohistochemical staining showing expression of IL-33 on murine RPE cells, original magnification ×100. (B) Expression of IL-33 and ST2 on murine RPE cells shown by flow cytometry. (C and D) *Il33* and *Mcp-1* mRNA expression by RPE cells analyzed by qPCR. Data are shown as mean + SEM of three samples from a single experiment. Data are representative of three independent experiments. *p < 0.05, **p < 0.01, ***p < 0.001, compared to medium alone as determined by Student's *t*-test.

It is well documented that many epithelial cells in tissues express IL-33 8. Retinal pigment epithelial (RPE) cells are an important component of the blood retina barrier, which is central to retinal homeostasis and involved in the development of many retinal diseases. We first examined the expression of IL-33 in an established murine RPE cell line 28. Cells cultured in cell chamber slides were used for immunohistochemical staining and cells cultured in flasks were harvested for flow cytometry analysis. Our data show that RPE cells expressed high levels of IL-33 as well as its receptor ST2 (Fig.1A and B). Furthermore, IL-33-activated RPE cells showed increased expression of *Il33* (Fig.1C) and *Mcp1* (Fig.1D) mRNA in an IL-33 dose-dependent manner although the effect of IL-33 was not as profound as LPS stimulation. These results demonstrate that IL-33 can amplify its own synthesis by the RPE cells, and suggest that IL-33 may have important roles in retinal homeostasis and disease development.

### IL-33 expression in naïve and EAU eye tissues

**Figure 2:**
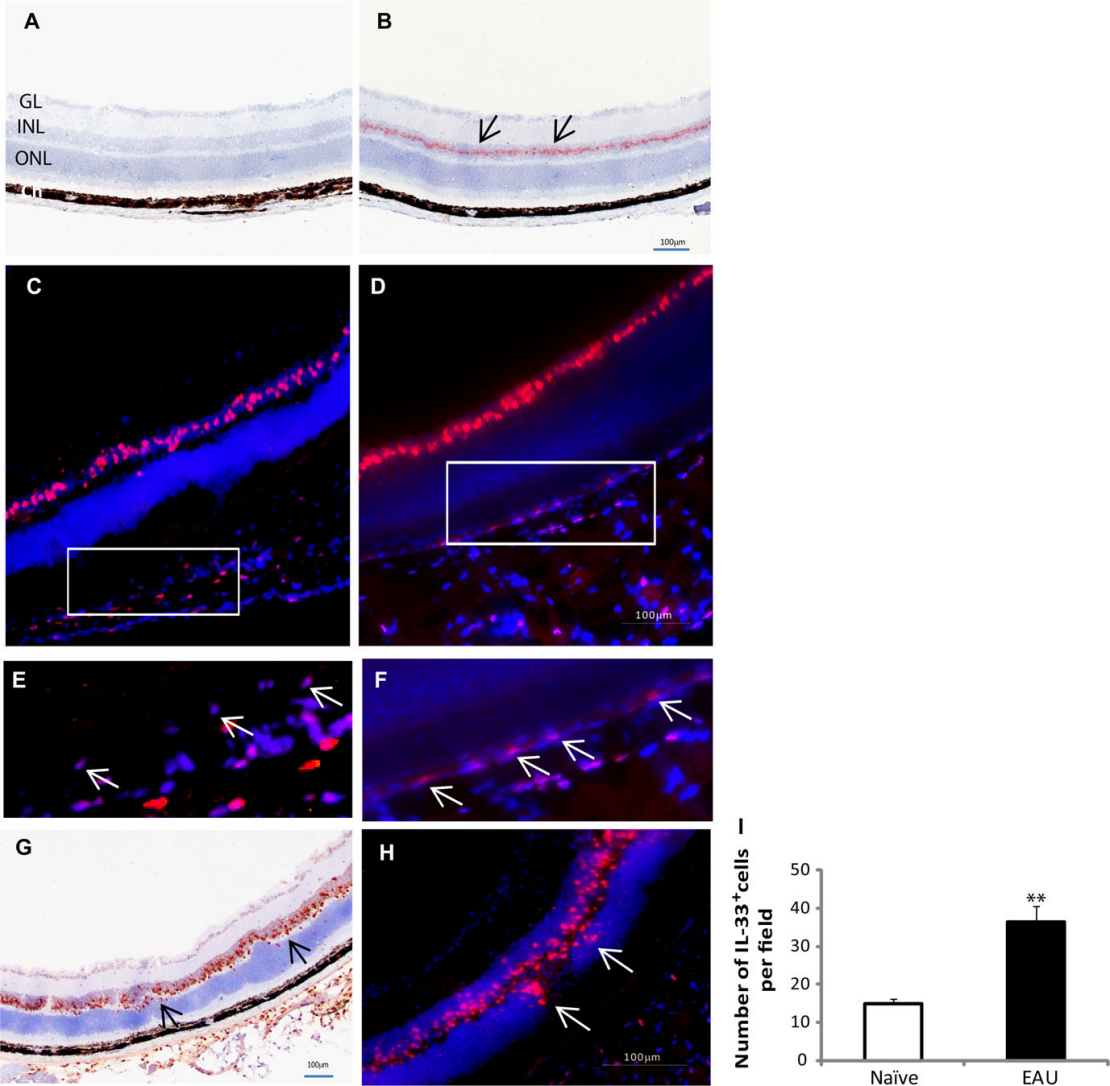
Expression of IL-33 in retinal tissues of naïve and EAU mice. (A) Isotype control staining in mouse retina; GL, ganglion cell layer; INL, inner nuclear layer; ONL, outer nuclear layer; Ch, choroid. (B) Immunohistochemical staining of IL-33 (AMEC, red) in the retina of naïve C57BL/6 mice, arrows indicate IL-33-positive cells. (C–F) Immunofluorescence staining of IL-33 expression (TRITC, red) in naïve retina of (C and E) C57BL/6 and (D and F) BALB/c mice, arrows in (E) and (F) indicate expression of IL-33 in RPE cells. (G) Immunohistochemical (DAB, brown) and (H) immunofluorescence (TRITC, red) staining of IL-33 expression in EAU retina tissues of C57BL/6 mice, arrows indicate IL-33-positive cells. Retinal images are representative of at least four mice per group. (I) Number of IL-33^+^ cells in each view field with 600× magnification of immunofluorescence-stained retina of naïve and EAU C57BL/6 mice was enumerated and shown as mean + SEM (*n* = 4 samples) and are pooled from two independent experiments. *p < 0.05, Student's *t*-test. Scale bars in the figures indicate 100 μm.

To identify the expression of IL-33 in vivo, we next examined the expression of IL-33 in the normal eye tissues as well as tissues from EAU mice. EAU was induced as described in “Materials and methods.” IL-33 was expressed by cells in the inner nuclear layer (INL) of retina tissues from naïve C57BL/6 mice (Fig.2A and B). Because of the dark pigment in the RPE cells and in the choroid in C57BL/6 mice, we used fluorescence immunohistochemical staining to study the expression of IL-33 in the retina of both C57BL/6 and BALB/c mice. Data in Figure2C and D confirmed IL-33 expression in INL cells in the retina of both strains of mice. Interestingly, IL-33 expression levels in the RPE cells were strain dependent, while RPE cells in C57BL/6 mice expressed low levels of IL-33 (Fig.2C and E), the expression was higher in the RPE cells of nonpigmented BALB/c mice (Fig.2D and F). In the retina tissue from EAU mice (day 16 postimmunization) of the C57BL/6 background, the expression of IL-33 was markedly increased in the INL with an increased number of IL-33 positive cells, and these cells moved toward the outer nuclear layer (Fig.2G–I). Similar levels of IL-33 expression were also observed in eye tissues harvested at day 21 after immunization. These results indicate that the expression of IL-33 is closely associated with EAU.

### Recombinant IL-33 treatment attenuate EAU development

**Figure 3:**
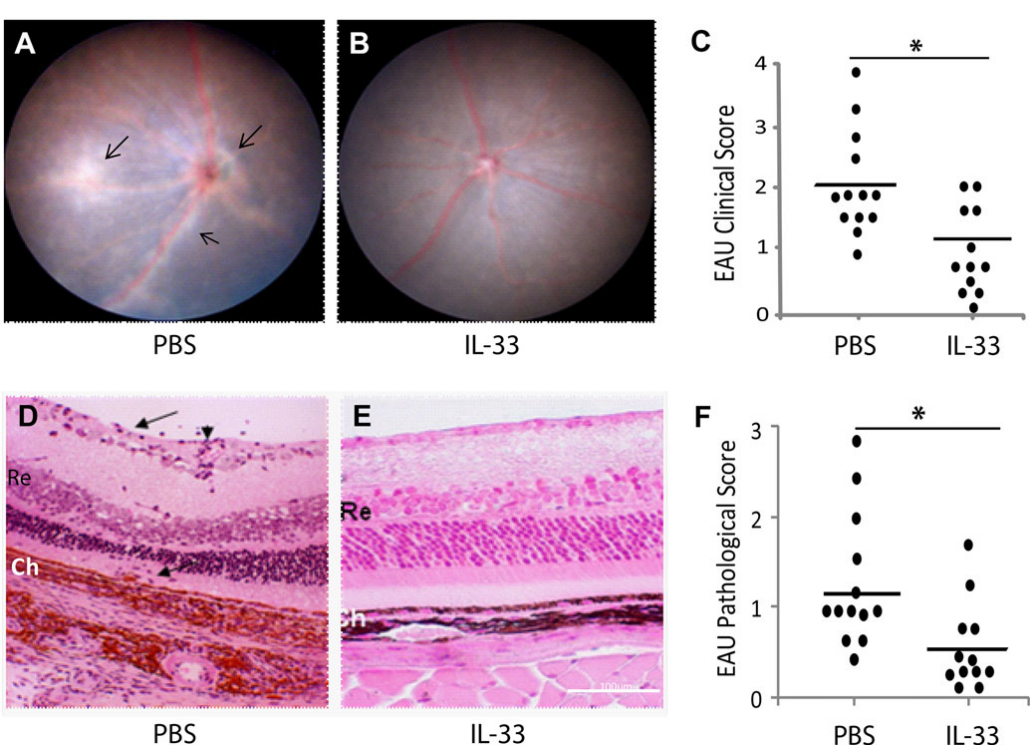
IL-33 attenuates EAU disease. C57BL/6 mice were immunized for EAU and injected i.p. with (0.5 μg/100 μL) IL-33 or 100 μL PBS every other day from day 9 to 25 postimmunization. Mice were examined for retinal inflammation using the fundus imaging system on day 18. (A and B) Representative images of retinal inflammation in (A) PBS or (B) IL-33-treated mice, arrows in (A) indicate vasculitis and exudates. (C) EAU clinical score of mice treated with PBS or IL-33. Mice were culled and eyes harvested on day 25 for histological examination. (D and E) Representative H&E staining images of retina of (D) PBS- or (E) IL-33-treated mice, arrows show infiltrating cells. Ch, choroid; Re, retina. (F) EAU pathological score of control and IL-33-treated mice. (C and F) Each symbol represents an individual mouse and bar indicates average score of EAU with *n* = 12 or 13 in each group. Data are representative of three independent experiments. *p < 0.05 as determined by Mann–Whitney test.

To directly assess the role of IL-33 in EAU development, we administered recombinant IL-33 i.p. to IRBP-immunized mice as described in the “Material and methods.” On day 18 after immunization, PBS-treated control mice developed retinal inflammation with swelling of the optic disc, diffuse whitish retinal exudates in the retina, and signs of vessel inflammation (vasculitis) (Fig.3A). In contrast, the retina of the IL-33-treated mice had significantly less inflammation (Fig.3B). IL-33-treated mice showed a significant reduction in the clinical score compared to the control PBS-treated mice (Fig.3C). The clinical findings were confirmed by histological examination of the eyes 25 days after immunization. Vasculitis and marked cell infiltration in the retina were observed in the retina of PBS-treated mice (Fig.3D), while minimal evidence of inflammation was seen in IL-33-treated mice (Fig.3E). IL-33-treated EAU mice developed significantly less retinal inflammation with reduced pathological scores (Fig.3F). Together, these results demonstrate a significant beneficial effect of IL-33 in EAU.

### ST2^−/−^ mice develop exacerbated EAU

**Figure 4:**
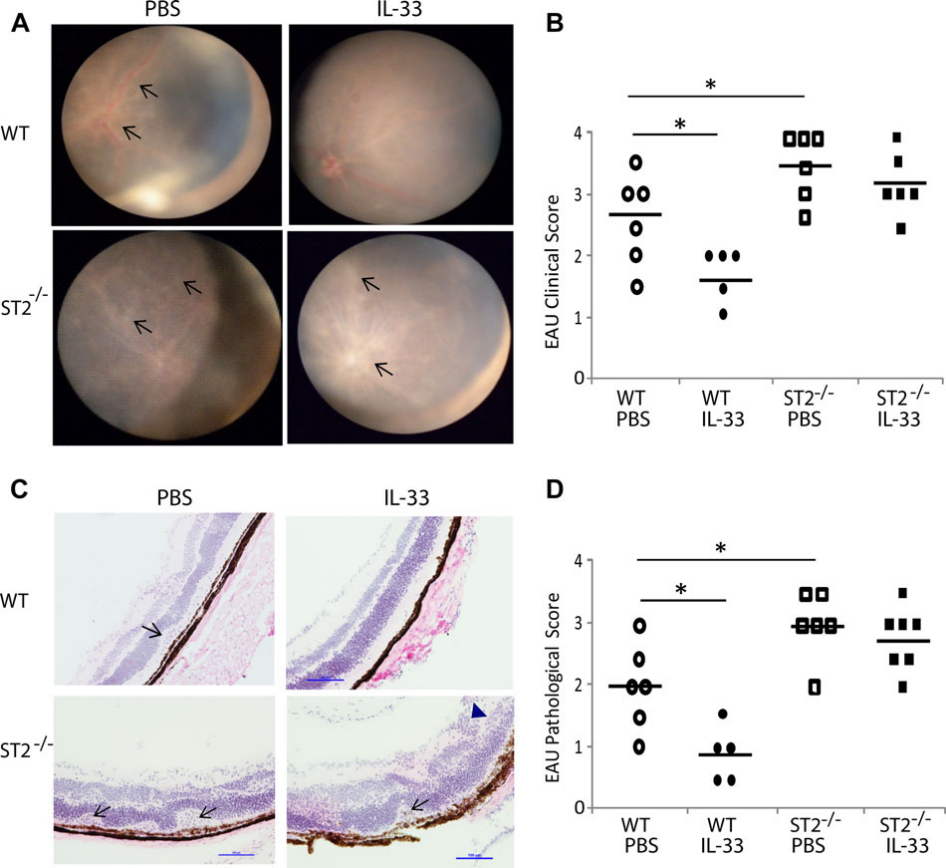
ST2^−^^/^^−^ mice developed exacerbated EAU. WT and ST2^−^^/^^−^ mice were immunized with 300 mg IRBP peptide_1–20_ and treated with PBS or IL-33 as in Figure3. Mice were examined for retinal inflammation clinically on day 20 and eye tissues examined for histological changes on day 22. (A) Retinal inflammation was examined using the fundus imaging system. Arrows indicate exudates and vasculitis in the retina. (B) EAU clinical scores of WT and ST2^−^^/^^−^ mice treated with PBS or IL-33. (C) Histological examination of the eye tissues stained with H&E staining, arrows indicate retinal detachment and infiltrating cells in retina (granuloma formation), arrowhead indicates vasculitis. (D) EAU pathological scores in WT and ST2^−^^/^^−^ mice. *p < 0.05 as determined by Student's *t*-test. Each symbol represents an individual mouse and bar indicates average score of EAU in each group (*n* = 5–7 mice) from a single experiment, which is representative of two independent experiments.

To identify an endogenous role of IL-33 in EAU, we next investigated the development of retinal inflammation in ST2**^−^**^/^**^−^** mice. WT and ST2**^−^**^/^**^−^** mice of the C57BL/6 background were immunized and then treated with PBS or IL-33 as above. Retinal inflammation was evaluated on day 20 after immunization using the fundus imaging system. As expected, WT mice developed optic disc swelling with diffuse and localized inflammation in the retina (Fig.4A, upper left panel). The disease was markedly more severe in the ST2**^−^**^/^**^−^** mice than in the WT mice. ST2**^−^**^/^**^−^** mice developed inflammation in the optic disc and surrounding retina, with vessels covered by inflammatory infiltrates and vessels in the optic disc barely visible (Fig.4A, lower left panel). Furthermore, while IL-33 treatment ameliorated the inflammation in the WT mice, IL-33 had no significant effect in the ST2**^−^**^/^**^−^** mice (Fig.4A, right panels). The corresponding clinical scores of the four groups of mice are shown in Figure4B. The clinical observation was further confirmed by histological analysis of the eye tissues on day 22 after immunization (Fig.4C and D). While infiltrating cells and vasculitis were seen in the retina of the control WT mice, minimal inflammation was observed in the retina tissue of IL-33-treated WT mice. In the ST2**^−^**^/^**^−^** mice, there was severe inflammation in the retina and choroid with retinal detachment, local folds, and massive infiltration. The disease in the ST2**^−^**^/^**^−^** mice was not attenuated by treatment with IL-33 (Fig.4D). These results therefore demonstrate an endogenous role of IL-33 in protecting mice against uveitis.

### IL-33 alters the cytokine production profile of leukocytes in EAU mice

**Figure 5:**
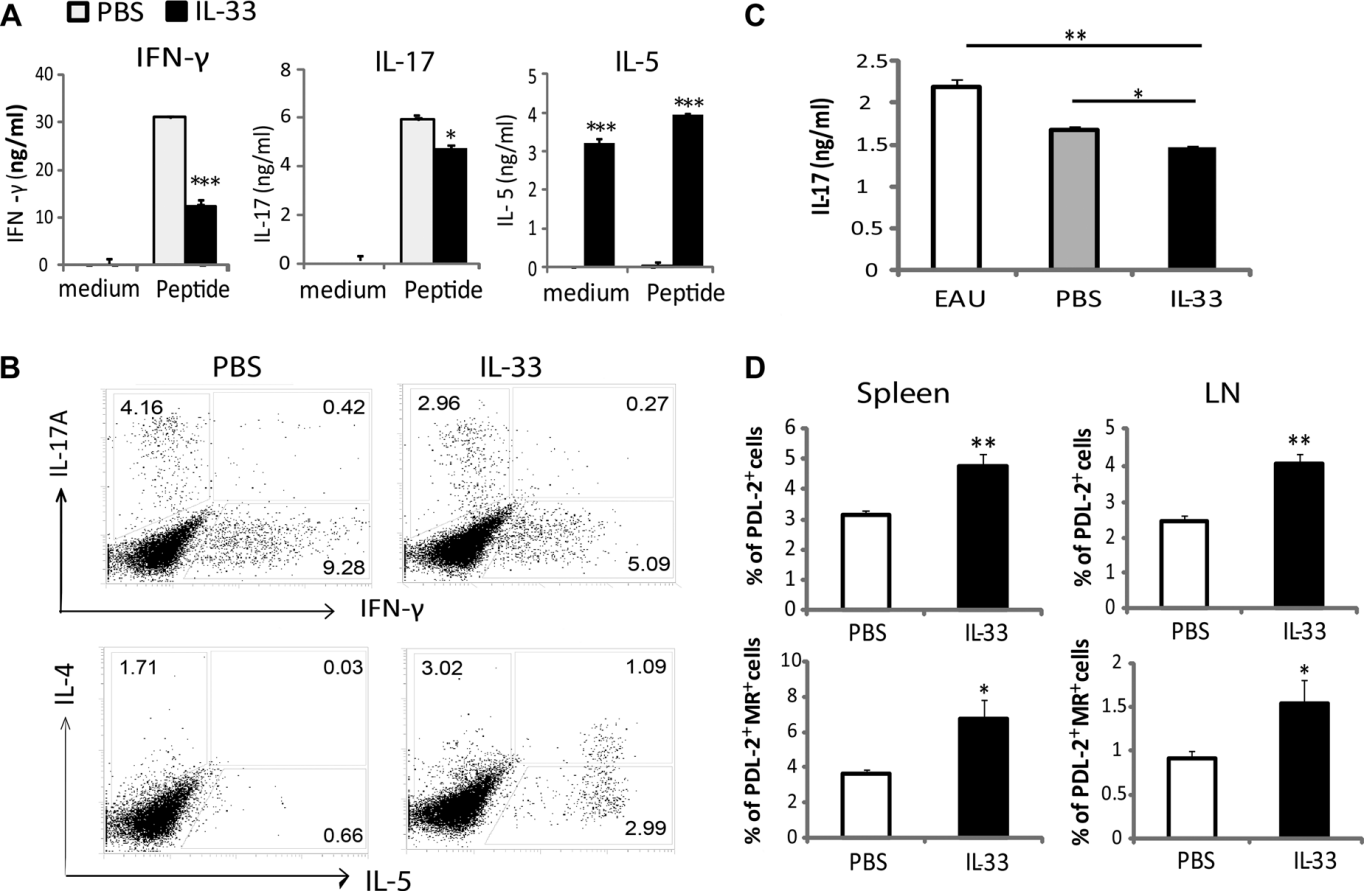
Cytokine production and cell phenotype in the lymphoid organs of EAU mice treated with IL-33. C57BL/6 mice were treated with IL-33 or PBS as in Figure3, and their DLNs and spleens collected on day 20 after immunization. (A) DLN cells were cultured with or without 50 μg/mL IRBP_1–20_ for 72 h and the concentrations of IFN-γ, IL-17, and IL-5 in the supernatant determined by ELISA. Data are shown as mean + SEM of pooled samples from five mice in each group from a single experiment, and are representative of three independent experiments. *p < 0.05, ***p < 0.001 compared to PBS-treated controls as determined by Student's *t*-test. (B) DLN cells from a pool of five mice per group were stained for intracellular cytokines. CD4^+^ cells were gated and analyzed for IL-17/IFN-γ or IL-4/IL-5 expression by flow cytometry. Data shown are representative of two experiments. (C) T cells purified from PBS or IL-33-treated mice were co-cultured with splenocytes of day 18 EAU mice in the presence of IRBP peptide_1–20_, and the concentration of IL-17 was determined by ELISA. Data are mean + SEM from four samples pooled from five mice in each group from a single experiment. *p < 0.05, **p < 0.01 compared to PBS control and EAU group as determined by Student's *t*-test. (D) F4/80^+^-gated DLN and spleen cells from PBS- or IL-33-treated mice were stained with anti-PD-L2 and anti-MR antibodies. Percentage of PD-L2^+^ or MR^+^/PD-L2^+^ double positive macrophages in the tissues was analyzed. Data are shown as mean + SEM with *n* = 7 from a single experiment, and are representative of two independent experiments. *p < 0.05, **p < 0.01 compared to PBS controls as determined by Student's *t*-test.

To investigate the immunological mechanisms underpinning the role of IL-33 in EAU, we examined the cytokine production by the DLN and spleen cells ex vivo. WT C57BL/6 mice were immunized and treated with IL-33 or PBS. Spleen and DLN cells were harvested on day 21 and single-cell suspensions were cultured with IRBP_1–20_ peptide. Supernatants were collected 72 h later and examined for cytokine production by ELISA. DLN cells from IL-33-treated EAU mice produced significantly less IFN-γ and IL-17 but more IL-5 compared to the cells from PBS-treated control mice (Fig.5A). Consistent with the cytokine data, there was also a significant reduction in the frequency of CD4^+^IL-17^+^, CD4^+^IFN-γ^+^, and CD4^+^IL-17^+^IFN-γ^+^ cells, but an increase in the percentage of CD4^+^IL-4^+^, CD4^+^IL-5^+^, and CD4^+^IL-4^+^IL-5^+^ T cells in the DLN (Fig.5B). Similar data were also observed in the spleen tissues (data not shown). Interestingly, IL-33 treatment did not lead to an increase in the frequency of CD25^+^ FoxP3^+^ regulatory T-cell population (data not shown). These results therefore demonstrate that IL-33 significantly switched a Th1 and Th17 response to the Th2 phenotype during EAU development. To further investigate whether T cells from IL-33-treated mice are able to suppress EAU-associated inflammation, we purified CD4^+^ T cells from PBS or IL-33-treated mice and co-cultured them with spenocytes from EAU mice in the presence of IRBP_1–20_ peptide. T-cell from IL-33-treated mice, but not PBS-treated mice, significantly suppressed IL-17 production by the spleen cells from EAU mice (Fig.5C).

### IL-33 treatment increases MR^+^PDL2^+^ M2 macrophage populations

Because of the well-defined role of IL-33 in macrophage polarization toward an M2 phenotype 29, and the potential protective role of M2 in tissue inflammation 30, we analyzed the macrophage phenotype in EAU mice with or without IL-33 treatment. WT C57BL/6 mice were immunized and treated with IL-33 or PBS as above and single-cell suspensions were prepared from the spleen and DLN tissues of mice 21 day after immunization. Cells were then stained for cell surface F4/80 (macrophage), CD206 (MR, mannose receptor) and CD273 (PD-L2), two markers closely identified with M2 31. Macrophages from IL-33-treated mice had significantly increased frequency of PD-L2^+^ and MR^+^/PD-L2^+^ cells in the LN and the spleen compared to that of the control PBS-treated mice (Fig.5D). These results therefore indicate that IL-33 treatment facilitates the polarization of M2, which may additionally account for the IL-33-mediated reduction of retina inflammation in EAU.

## Discussion

Data presented here demonstrate that IL-33 has a protective effect on intraocular inflammation in EAU. In mice lacking the IL-33 receptor ST2, EAU was more severe while treatment of mice with IL-33 attenuated disease severity in WT mice but not in the ST2**^−^**^/^**^−^** mice. The protective effect of IL-33 in EAU was associated with a shift of immune responses toward type 2 immune responses as demonstrated by the increased frequency of Th2 cells and M2 in the lymphoid organs.

IL-33 is constitutively expressed in normal epithelial and endothelial cells in tissues such as human colon, lung, liver, and skin 8,16, as well as in human vascular endothelial cells in allergic papillary conjunctivitis 32. We show here the presence of IL-33 and ST2 in cultured RPE cells and demonstrated an autocrine upregulation of IL-33 in these cells. Many RPE-derived molecules are known to be involved in local immune privilege in the eye 33; our findings here suggest that IL-33 may also be a key immune-regulatory mediator in this context. LPS induced significantly higher expression of both *Il33* and *Mcp1* mRNA suggesting that the induction of EAU with CFA may well be a competitive balance between pro- and anti-inflammatory signals of innate immunity. We also found that the levels of IL-33 expression in the RPE cells in retina tissues vary in different strains of mice. While IL-33 is highly expressed by the RPE cells in the naïve BALB/c mice, the expression level was low in these cells in the naïve C57BL/6 background mice. This observation is consistent with a protective role of IL-33 in retinal inflammation as BALB/c mice are resistant whereas C57BL/6 mice are susceptible to EAU 34,35. Further immunohistochemical staining in the retina of naïve mice shows that that IL-33 was also constitutively expressed by cells in the INL of retina, suggesting IL-33 may have eye-specific functions under homeostasis and disease conditions. Our data of IL-33 staining in the INL also agree with a recent report that used an IL-33-LacZ gene trap reporter 36. The author further suggested that the IL-33-positive cells are likely to be Muller glial cells. Since microglia cells also exist in the INL of retina, it would be interesting to confirm whether they also express IL-33 in the retina tissues. IL-33 expression in the retina was elevated with increased numbers of IL-33^+^ cells moved toward the outer nuclear layer during EAU. These data indicate that IL-33 is closely associated with the posterior inflammation of the eye, and hence might contribute to its immune regulatory role in ocular inflammation. The elevated expression of IL-33 in the retina of EAU mice may represent a failed attempt by the mice to contain EAU, which can be effectively ameliorated with additional exogenously administered recombinant IL-33.

IL-33 plays an important role in many inflammatory diseases but its effect is model- and disease-specific 17. While IL-33 exacerbates autoimmune collagen-induced arthritis via mast cell degranulation 22, it also promotes type 2 immune responses with the production of high levels of IL-5 and IL-13 20 and drives M2 macrophage differentiation thus protecting mice from EAE 26 and atherosclerosis 20. However, by doing so, IL-33 contributes to the pathogenesis of asthma, atopic dermatitis, and allergic conjunctivitis 18,27. Data reported here suggest that IL-33 is protective against retinal inflammation, and the effect is likely to be endogenous as ST2^−/−^ mice developed more severe EAU.

Although the relative importance of Th1 and Th17 cells in the development of autoimmunity is still debated, the current view suggests a more complex relationship between the two cell subsets; they have overlapping and differential roles 1 in the pathogenesis of EAU and other autoimmune diseases such as EAE 37. Classically activated macrophages are often involved in tissue damage during early and peak stages of inflammatory diseases, while regulatory M2 macrophages that appear in late stages can significantly limit the damage 29,30,38. It is generally agreed that a shift in T-cell and cytokine profile toward a type 2 phenotype and the emergence of regulatory T cells 39–41 are responsible for disease protection in EAE and EAU 5,42. While we did not observe an apparent increase of FoxP3^+^ Treg cell population in the lymphoid organs of IL-33-treated mice, it is likely that IL-33 reduces retinal inflammation via the reduction of Th1 and Th17 cells and skewing the immune response toward noninflammatory, protective type 2 responses. As IL-33 is also important in activating antigen presenting cells 43,44 or innate lymphoid cells 45 to induce Th2 type responses, it may have attenuated the severity of EAU through inhibiting both the innate and the adaptive immune responses, e.g. switching the phenotype of antigen presenting DCs and macrophages in the peripheral lymphoid organs, and the microglia cells in the target retina.

EAU shares many features of clinical uveitis. Data presented here that IL-33 plays an endogenous tissue protective role in EAU and IL-33 administration ameliorates EAU, suggest that IL-33 may be a potential option for treating clinical uveitis.

## Materials and methods

### Mice and reagents

C57BL/6 and BALB/c mice were purchased from Harlan (UK) and maintained at the Biological Procedure Unit, University of Strathclyde and University of Aberdeen. ST2**^−^**^/^**^−^** mice on C57BL/6 background were described previously 20. ST2**^−^**^/^**^−^** and the WT littermates were maintained in the animal facilities of Glasgow University. All experiments were performed under the guidelines of the UK Home Office Animals (Scientific Procedures) Act 1986 and to the Association for Research in Vision and Ophthalmology Statement for the Use of Animals in Ophthalmic and Vision Research. Female mice (7–12 weeks old) were used in all experiments. Murine recombinant IL-33 was obtained from BioLegend (UK). 

### RPE cell culture and qPCR

RPE cell line (B6-RPE07) was derived from a 12-week-old naïve female C57BL/6 mouse and generated as described previously 28. Cells were cultured in T75 flasks in complete DMEM containing 10% (v/v) fetal calf serum (Sigma) and incubated in 5% CO_2_ at 37°C. Seventy percent confluent cells were detached from flasks using 0.25% trypsin-EDTA and cultured in 24-well plates with different concentrations of IL-33 (10–100 ng/mL) or LPS (100 ng/mL, Sigma) for 24 h. Cells were then collected and lysed with RNA lysis buffer (Ambion, Life Technologies) for analysis of *Il33* and *Mcp-1* mRNA expression using Fast SYBR® Green real time PCR.

### EAU induction and clinical evaluation

EAU was induced as described previously 3. In brief, WT or ST2**^−^**^/^**^−^** C57BL/6 mice were immunized subcutaneously on the lower back with 300 mg of IRBP_1–20_ peptide (Sigma Genosys) in 50 μL of PBS emulsified with an equal volume of CFA (4 mg/mL *Mycobacterium tuberculosis*, strain H37RA, Difco, Detroit MI). Each mouse also received i.p. 500 ng/200 mL of pertussis toxin (PTX, Sigma) in PBS. To investigate the effect of IL-33 on EAU development, WT mice were injected i.p. every other day with IL-33 (0.5 μg/100 μL) from day 9 to 25 after the initial immunization. Control WT mice received the same volume of PBS. Clinical evaluation of ocular inflammation was performed using the fundus imaging system as previously described 46. Briefly, mice were anesthetized and pupils were dilated before taking images, and viscoelastic material was used as medium for corneal surface during imaging.

### Histological evaluation of disease

Mice were euthanized in CO_2_ chamber and their eyes were carefully removed and fixed in 2.5% (w/v) glutaraldehyde and embedded in resin for standard H&E staining. In some experiments, removed eyes were snap frozen in tissue freezing optimum cutting temperature medium on dry ice for cryosectioning, H&E or immunohistochemical staining.

### Immunohistochemical staining

RPE cells were cultured in cell chambers and stained with anti-IL-33 antibody (R&D Systems). Eye tissues of naïve and EAU mice (day 16 after immunization) were frozen in optimum cutting temperature and 8 μm sections were stained with anti-IL-33 antibody, followed by incubation with biotinylated secondary antibody (Vector Laboratories) and detected with substrates (DAB or AMEC from Vector Laboratories). For fluorescence staining, TRITC-conjugated strepavidin was added to the tissues sections following incubation with the secondary antibody. Fluorescence staining sections were mounted with Vectashield containing DAPI (Vector Laboratories). Isotypes with matching IgG (R&D Systems) were used as negative controls.

### DLN and spleen cell culture

DLN and spleen were collected and pooled within groups or analyzed individually. Single-cell suspensions were cultured in 24-well plates at 4 × 10^6^ cells/2 mL per well and stimulated with medium alone or with 50 μg/mL of IRBP_1–20_ peptide. After 72 h, supernatants were collected and concentrations of selected cytokines were measured by ELISA using paired antibodies (eBioscience).

### T-cell–EAU splenocyte co-culture

Mice were treated with five doses of IL-33 (0.5 μg/100 μL) or PBS every second day. Spleen and LNs were harvested and pooled within each group. Single-cell suspensions were collected and CD4^+^ T cells were purified using CD4^+^ T-cell isolation kit II (Miltenyi Biotec, UK). Purified T cells (1 × 10^6^) were co-cultured (in 24-well pates, 2 mL per well) with 2 × 10^6^ spenocytes of day 18 EAU mice in the presence of 50 μg/mL IRBP peptide. After 72 h, supernatants were harvested for IL-17 ELISA.

### Flow cytometry analysis

Antibodies were purchased from BD Pharmingen unless mentioned otherwise. RPE cells were harvested and washed before staining with anti-IL-33 (R&D System) or ST2 (MD Biosciences) antibodies. To examine the leukocyte phenotype in the DLN and spleen, single-cell suspensions were stained with anti-F4/80, anti-CD206 (MR, Serotec) and anti-CD273 (PD-L2, eBioscience) antibodies. For measurement of intracellular cytokines, cells were restimulated with 50 ng/mL phorbol-12-myristate-13-acetate and 500 ng/mL ionomycin (both from Sigma) in the presence of Golgi-Stop (BD Bioscience) for 4 h. Cells were then harvested and stained with cell surface anti-CD4 antibody before being permeabilized with Perm/Fix solution (eBioscience) and finally stained with anti-IL-17A (clone eBio17B7), anti-IFN-γ (clone XMG1.2), anti-IL-5 (TRFK4), and anti-IL-4 (clone 11B11) antibodies (eBioscience). Isotype-matched IgG antibodies from eBioscience were used as negative controls. Cells were analyzed by FACS Calibur using CellQuest software (BD Biosciences).

### Statistical analysis

Statistical evaluations of cell frequency, cytokine production, and histological analysis were performed with the two-tail unpaired Student's *t*-test or Mann–Whitney test. *p* < 0.05 was considered statistically significant. All experiments were repeated at least twice.

## Abbreviations

DLN: draining lymph node
EAU: experimental autoimmune uveitis
IL-33: Interleukin-33
INL: inner nuclear layer
M2: alternatively activated macrophage
MR: mannose receptor
RPE: retinal pigment epithelial

## Supporting Information

As a service to our authors and readers, this journal provides supporting information supplied by the authors. Such materials are peer reviewed and may be re-organized for online delivery, but are not copy-edited or typeset. Technical support issues arising from supporting information (other than missing files) should be addressed to the authors.

Peer review correspondence
