# Supplementary material for: IL-33 attenuates the development of experimental autoimmune uveitis
Source: Eur J Immunol. 2014 Oct 18;44(11):3320–9. doi: 10.1002/eji.201444671 (PMC4449115; doi:10.1002/eji.201444671)
Supplement: Supplementary file 1 — Peer review correspondence [file eji0044-3320-sd1.pdf]

European Journal of Immunology

# Peer review correspondence

## IL-33 attenuates the development of experimental autoimmune uveitis

Mark Barbour, Debbie Allan, Heping Xu, Cheng Pei, Mei Chen, Wanda Niedbala, Sandra Y. Fukada, Anne-Galle Besnard, Jose C Alves-Filho, Xiaoguang Tong, John V. Forrester, Foo Yew Liew and Hui-Rong Jiang

Corresponding author: Hui-Rong Jiang, Strathclyde Institute of Pharmacy and Biomedical Sciences, University of Strathclyde, Glasgow, United Kingdom

---

| Review Timeline: | Submission date: | 19 March 2014 |
|---|---|---|
| | Editorial decision: | 23 April 2014 |
| | Revision received: | 25 June 2014 |
| | Accepted: | 8 August 2014 |

---

Handling Executive Committee member: Dr. Steffen Jung

Please note that the correspondence below does not include the standard editorial instructions regarding preparation and submission of revised manuscripts, only the scientific revisions requested and addressed.

First Editorial Decision – 23 April 2014

Dear Dr. Jiang,

Manuscript ID eji.201444671 entitled "IL-33 attenuates the development of experimental autoimmune uveitis" which you submitted to the European Journal of Immunology has been reviewed. The comments of the referees are included at the bottom of this letter.

A revised version of your manuscript that takes into account the comments of the referees will be reconsidered for publication. You will see that although your submission received a very positive review from referee 1, referee 2 felt that the novelty of the findings needs to be expanded with more experimental data. Furthermore, referee 3 feels that some of your conclusions are not supported by the data shown, especially in light of the system/methodology under which the results were obtained. We therefore strongly encourage you to fully address all of the points of referees 1 and 3 and to incorporate as much of referee 2's suggestions as appropriate to the revision. We also ask that you tone down some of your conclusions as alluded to by referee 3.

You should also pay close attention to the editorial comments included below. *In particular, please edit your figure legends to follow Journal standards as outlined in the editorial comments. Failure to do this will result in delays in the re-review process.*

Please note that submitting a revision of your manuscript does not guarantee eventual acceptance, and that your revision will be re-reviewed by the referees before a decision is rendered.

If the revision of the paper is expected to take more than three months, please inform the editorial office. Revisions taking longer than six months may be assessed by new referees to ensure the relevance and timeliness of the data.

Once again, thank you for submitting your manuscript to European Journal of Immunology and we look forward to receiving your revision.

Yours sincerely,
Karen Chu

On behalf of Dr. Steffen Jung

Dr. Karen Chu
Editorial Office
European Journal of Immunology
e-mail: ejied@wiley.com
www.eji-journal.eu

**********************************************************

Reviewer: 1

Comments to the Author
This study extends the knowledge concerning the immunomodulating activities of IL-33. Testing it in the eye system yielded new interesting pieces of information. The study has been well done and clearly recorded.

A minor critical comment: It would be useful if the Figures showing clinical and histological ocular changes would be accompanied by the corresponding scores.

# Peer review correspondence

European Journal of Immunology

Reviewer: 2

Comments to the Author

The authors studied the role of IL-33 and its receptor in the development of experimental autoimmune uveitis (EAU). They reported that IL-33 and IL-33 receptor (ST2) were expressed in murine retinal cells; ST2-deficient mice developed exacerbated EAU, whereas IL-33 significantly reduced EAU; the attenuated EAU in IL-33-treated mice was accompanied by decreased IFN-γ+ and IL-17+ CD4+ T cells and increased IL-5+ and IL-4+ CD4 T cells; and macrophages from the IL-33-treated mice polarized towards M2 phenotype.

The paper is clearly written. Although this is the first report about IL-33 in EAU, the novelty needs to be enhanced by the experiments such as:

Whether IL-33 affects systemic and/or local infiltrating macrophages,

Whether IL-33 or IL-33R expressing RPE have direct effects on macrophages and T cells

Whether IRBP-specific T cells derived from IL-33 treated or ST2 KO EAU mice could induce EAU after transferring them into naïve mice,

Whether IRBP-specific T cells derived from IL-33 treated or ST2 KO EAU mice either inhibit or enhance IRBP-specific T cells from WT mice to induce EAU in naive mice,

Whether macrophages from IL-33 treated EAU mice could convert IRBP-specific effector T cells into Th2 and/or generate more Th2.

Reviewer: 3

Comments to the Author

The authors demonstrate that injections of IL-33, a cytokine seen expressed in the healthy retina, suppress EAU by altering the cytokine pattern the suggests induction of Th2 cells. While the authors correctly propose that using IL-33 can suppress the severity of EAU they are far from explaining how it works. IL-33 is injected early in the induction of EAU, a time that probably cannot be matched in humans, and it is injected systemically. The potential that it mediates the activation of M2 APC is interesting since others (not cited by the authors) and by the authors have shown that microglial cells in the retina appear to be alternatively activated. The authors for balance should include in the discussion the influence that IL-33 will have on APC processing and presentation of antigen. Since the injection of IL-33 is at the start of the affecter phase immunity it is very well possible that what is being observed is suppression of innate immune signals, not necessarily the conversion of APC or T cells.

European Journal of Immunology

1. In figure 1 C and D the dose dependent manner appear to be with only Mcp1 mRNA and not with Il33. This should be corrected in the results or more data presented. Also, add a positive stimulating control for Mcp1 mRNA. It results suggest that IL-33 may have some effect on augmenting the mRNA levels of IL-33 itself and Mcp-1, it is not as effective as a TLR4 stimulator. Since adjuvant is important in the immunization for EAU, the inability of IL-33 to be as strong as LPS suggests that the induction of EAU may well be a competitive balance pro and anti-inflammatory signal of innate immunity.

2. In figure 2. It would help if co-staining were done on the retinal sections. Since microglial cells occupy the inner nuclear layer, are the IL-13 cells microglial cells or the muller cells? If the muller cells then why is the staining limited to only the INL. Their levels of IL-33 expression need to be quantified. Also, in Figure 2 G and h, at what time in EAU was this section assayed and doe the levels change with the course of disease. Since EAU is a limited disease in mice, does IL-33 increase in the resolution?

3. Figure 5. To full demonstrate that these converted APC or T cells can suppress or no longer mediate inflammation they need to be sorted and adoptively transferred either into EAU immunized mice to show suppression of EAU or used a local adoptive transfer assay to show suppressed inflammatory response by the T cells. Also, flow on FoxP3 staining need to be included. Also, better compensation is needed for the analysis in Figure 5B.

4. The results of the flow in Figure 5B do not correspond with the cytokine measured in figure 5A. In 5A the levels of IL-17 are marginally suppressed, but in the flow there is almost a 50% loss in IL-17 producing cells, in contrast IFN-γ production is more than 66% suppressed in 5A but only 47% in 5B. Also there is no measurement of IL-4 in Figure 5A. This experiment would be better if flow or cytokine production was the focus along with an adoptive transfer of cells to show their corresponding function. Also, the flow on unstimulated T cells is a necessary control to demonstrate antigen-specific changes.

5. In the discussion, the authors make the following claim:
…it is generally agreed that a shift in T cell and cytokine profile towards a type 2 phenotype is responsible for disease protection in EAE and EAU [5, 38].
This is not true. There is no general agreement that a shift to type 2 phenotype is responsible at all for disease protection in EAU.
See for example:
1. Blockade of interleukin-6 signaling suppresses not only th17 but also interphotoreceptor retinoid binding protein-specific Th1 by promoting regulatory T cells in experimental autoimmune uveoretinitis. Haruta H, Ohguro N, Fujimoto M, Hohki S, Terabe F, Serada S, Nomura S, Nishida K, Kishimoto T, Naka T. Invest Ophthalmol Vis Sci. 2011 May 17;52(6):3264-71. PMID: 21330657
2. Zhou R, Horai R, Silver PB, Mattapallil MJ, Zárate-Bladés CR, Chong WP, Chen J, Rigden RC, Villasmil R, Caspi RR. The living eye "disarms" uncommitted autoreactive T cells by converting them to

European Journal of Immunology

Foxp3(+) regulatory cells following local antigen recognition. J Immunol. 2012 Feb 15;188(4):1742-50. PubMed PMID: 22238462; PubMed Central PMCID: PMC3273602.

3. Sun M, Yang P, Du L, Zhou H, Ren X, Kijlstra A. Contribution of CD4+CD25+ T cells to the regression phase of experimental autoimmune uveoretinitis. Invest Ophthalmol Vis Sci. 2010 Jan;51(1):383-9. PubMed PMID: 19696173.

First revision – authors' response – 25 June 2014

Reviewer 1:

This study extends the knowledge concerning the immunomodulating activities of IL-33. Testing it in the eye system yielded new interesting pieces of information. The study has been well done and clearly recorded.

A minor critical comment: It would be useful if the Figures showing clinical and histological ocular changes would be accompanied by the corresponding scores.

Reply - We have now added the corresponding clinical scores for ocular changes (Figure 4B). Details are also added in the text (page 9).

Reviewer: 2

The authors studied the role of IL-33 and its receptor in the development of experimental autoimmune uveitis (EAU). They reported that IL-33 and IL-33 receptor (ST2) were expressed in murine retinal cells; ST2-deficient mice developed exacerbated EAU, whereas IL-33 significantly reduced EAU; the attenuated EAU in IL-33-treated mice was accompanied by decreased IFN-γ+ and IL-17+ CD4+ T cells and increased IL-5+ and IL-4+ CD4 T cells; and macrophages from the IL-33-treated mice polarized towards M2 phenotype.

The paper is clearly written. Although this is the first report about IL-33 in EAU, the novelty needs to be enhanced by the experiments such as: Whether IL-33 affects systemic and/or local infiltrating macrophages, Whether IL-33 or IL-33R expressing RPE have direct effects on macrophages and T cells Whether IRBP-specific T cells derived from IL-33 treated or ST2 KO EAU mice could induce EAU after transferring them into naïve mice, Whether IRBP-specific T cells derived from IL-33 treated or ST2 KO EAU mice either inhibit or enhance IRBP-specific T cells from WT mice to induce EAU in naive mice, Whether macrophages from IL-33 treated EAU mice could convert IRBP-specific effector T cells into Th2 and/or generate more Th2.

Reply - We agree with the reviewer that it would be of interest to conduct further investigations, however, we feel that this series of experiments could be a subject of future studies.

Reviewer 3:

The authors demonstrate that injections of IL-33, a cytokine seen expressed in the healthy retina, suppress EAU by altering the cytokine pattern the suggests induction of Th2 cells. While the authors correctly propose that using IL-33 can suppress the severity of EAU they are far from explaining how it works. IL-33 is injected early in the induction of EAU, a time that probably cannot be matched in humans, and it is injected systemically. The potential that it mediates the activation of M2 APC is interesting since others (not cited by the authors) and by the authors have shown that microglial cells in the retina appear to be alternatively activated. The authors for balance should include in the discussion the influence that IL-33 will have on APC processing and presentation of antigen. Since the injection of IL-33 is at the start of the affecter phase immunity it is very well possible that what is being observed is suppression of innate immune signals, not necessarily the conversion of APC or T cells.

Reply - We thank the reviewer for his/her comments about the potential effect of IL-33 on EAU development via APC activation. We have now included in the discussion about the influence IL-33 may have on APC cells during the development of EAU (page 13, line 6-10).

1. In figure 1 C and D the dose dependent manner appear to be with only Mcp1 mRNA and not with Il33. This should be corrected in the results or more data presented. Also, add a positive stimulating control for Mcp1 mRNA. It results suggest that IL-33 may have some effect on augmenting the mRNA levels of IL-33 itself and Mcp-1, it is not as effective as a TLR4 stimulator. Since adjuvant is important in the immunization for EAU, the inability of IL-33 to be as strong as LPS suggests that the induction of EAU may well be a competitive balance pro and anti-inflammatory signal of innate immunity.

Reply - We have added a positive stimulating control for Mcp-1 mRNA (Figure 1D) and also presented the data in a consistent format in Figure 1C and 1D. We thank the reviewer for his comments “that IL-33 may have some effect on augmenting the mRNA levels of IL-33 itself and Mcp-1, it is not as effective as a TLR4 stimulator. …”, we have modified the legend and the manuscript accordingly (page7 and 11-line 13-16).

2. In figure 2. It would help if co-staining were done on the retinal sections. Since microglial cells occupy the inner nuclear layer, are the IL-13 cells microglial cells or the muller cells? If the muller cells then why is the staining limited to only the INL. Their levels of IL-33 expression need to be quantified. Also, in Figure 2 G and h, at what time in EAU was this section assayed and doe the levels change with the course of disease. Since EAU is a limited disease in mice, does IL-33 increase in the resolution?

Reply - We have performed dual immunohistochemical staining with IL-33 and microglia (Iba-1 Ab purchased from Abcam). Although our data (Images below) show that IL-33 co-localises with Iba-1 molecule in the INL, the staining of Iba-1 did not show ramified morphology of microglia cells. We have discussed these data with Professor H Xu (Queens University Belfast) as his lab has performed many IHC staining of microglia cells in retina. Interestingly his lab also observed similar staining of Iba-1 (the same Abcam Ab) in INL, however he thinks that the microglia staining with this Iba-1 Ab is not convincing without showing ramified morphology, and furthermore his lab data also suggested that IL-33 is likely co-localised with Muller cells. For this study, we have now modified our manuscript to reflect this (page 11, bottom line and page 12).

The EAU section in Figure 2 was collected from mice at day 16 post immunisation. This information is now included in the manuscript (page 8, 1st line). We also performed more immunohistochemical staining on day 21 EAU eye tissues, and our data showed similar increased level of IL-33 staining compared with day 16 and the information is added in the text (page 8, line 3-4).

- We agree that quantification of the fluorescence would provide quantitative information on the level of up-regulation of IL-33 in EAU retina. We counted IL-33 expressing cells in each view field with 600x magnification. Our data show that the increase of IL-33+ cell number in the EAU retina tissue is obvious, the information is now included in Figure 2 I and page 8 (line 2-3).

3. Figure 5. To full demonstrate that these converted APC or T cells can suppress or no longer mediate inflammation they need to be sorted and adoptively transferred either into EAU immunized mice to show suppression of EAU or used a local adoptive transfer assay to show suppressed inflammatory response by the T cells. Also, flow on FoxP3 staining need to be included. Also, better compensation is needed for the analysis in Figure 5B.

Reply - In another autoimmune disease model, we previously purified type 2 macrophages of IL-33 treated mice and were able to show that these M2 macrophages partially inhibited the development of autoimmune disease (ref 26). We have now investigated the effect of CD4+ T cells on IL-17 production by EAU spenocytes. Purified CD4 T cells from IL-33 treated mice reduced the production of IL-17 by EAU mice spleen cells. These new data are now included in Figure 5C and in the text (page 10, 16).

- We also examined the % of CD4+FOXP3+ cells in the CD4+ cell population in both spleen and draining lymph nodes (figure below, mean□SD). There was no significant difference between IL-33 and PBS-treated mice (two experiments). The compensation was set up according to isotype control.

4. The results of the flow in Figure 5B do not correspond with the cytokine measured in figure 5A. In 5A the levels of IL-17 are marginally suppressed, but in the flow there is almost a 50% loss in IL-17 producing cells, in contrast IFN-γ production is more than 66% suppressed in 5A but only 47% in 5B. Also there is no

European Journal of Immunology

measurement of IL-4 in Figure 5A. This experiment would be better if flow or cytokine production was the focus along with an adoptive transfer of cells to show their corresponding function. Also, the flow on unstimulated T cells is a necessary control to demonstrate antigen-specific changes.

Reply - There is some misunderstanding in the data of Figure 5. Fig 5A measures the concentrations of cytokines produced by immune cells in culture with or without MOG peptide (antigen specific) whereas Fig 5B measures the percentage of cytokine secreting CD4 cells after in vitro stimulating with phorbol-12-myristate-13-acetate and ionomycin for 4 hours (this is a common protocol to detect the percentage of cytokine secreting cells using FACS, so it is not an antigen-specific stimulation). Data from ELISA (5A) and FACS (5B) show same trend, but not comparable as they are two different measurements on different parameters. IL-4 concentration was below detectable level by ELISA (e-Bioscience reagents).

5. In the discussion, the authors make the following claim:
…it is generally agreed that a shift in T cell and cytokine profile towards a type 2 phenotype is responsible for disease protection in EAE and EAU [5, 38]. This is not true. There is no general agreement that a shift to type 2 phenotype is responsible at all for disease protection in EAU. See for example:
1. Blockade of interleukin-6 signaling suppresses not only th17 but also interphotoreceptor retinoid binding protein-specific Th1 by promoting regulatory T cells in experimental autoimmune uveoretinitis. Haruta H, Ohguro N, Fujimoto M, Hohki S, Terabe F, Serada S, Nomura S, Nishida K, Kishimoto T, Naka T. Invest Ophthalmol Vis Sci. 2011 May 17;52(6):3264-71. PMID: 21330657 2. Zhou R, Horai R, Silver PB, Mattapallil MJ, Zárate-Bladés CR, Chong WP, Chen J, Rigden RC, Villasmil R, Caspi RR. The living eye "disarms" uncommitted autoreactive T cells by converting them to Foxp3(+) regulatory cells following local antigen recognition. J Immunol. 2012 Feb 15;188(4):1742-50. PubMed PMID: 22238462; PubMed Central PMCID: PMC3273602.
3. Sun M, Yang P, Du L, Zhou H, Ren X, Kijlstra A. Contribution of CD4+CD25+ T cells to the regression phase of experimental autoimmune uveoretinitis. Invest Ophthalmol Vis Sci. 2010 Jan;51(1):383-9. PubMed PMID: 19696173.

Reply - We thank the reviewer for pointing out the important roles of Treg cells in EAU resolution stage, we have now revised our manuscript accordingly and added the relevant references (page 13, line 1-4).

<u>Second Editorial Decision – 28 July 2014</u>

Dear Dr. Jiang,

It is a pleasure to provisionally accept your manuscript entitled "IL-33 attenuates the development of experimental autoimmune uveitis" for publication in the European Journal of Immunology. For final

European Journal of Immunology

acceptance, please follow the instructions below and return the requested items as soon as possible as we cannot process your manuscript further until all items listed below are dealt with.

Please note that EJI articles are now published online a few days after final acceptance (see Accepted Articles: http://onlinelibrary.wiley.com/journal/10.1002/(ISSN)1521-4141/accepted). The files used for the Accepted Articles are the final files and information supplied by you in Manuscript Central. You should therefore check that all the information (including author names) is correct as changes will NOT be permitted until the proofs stage.

We look forward to hearing from you and thank you for submitting your manuscript to the European Journal of Immunology.

Yours sincerely,
Karen Chu

on behalf of Dr. Steffen Jung

Dr. Karen Chu
Editorial Office
European Journal of Immunology
e-mail: ejied@wiley.com
www.eji-journal.eu
